# Study of seminal infection among an infertile male population in Qom, Iran, and its effect on sperm quality

**Published:** 2018-04

**Authors:** Katayon Berjis, Mahdieh Ghiasi, Sareh Sangy

**Affiliations:** 1 Mirza Koochak Khan Infertility Center, Tehran University of Medical Sciences, Tehran, Iran; 2 Department of Pharmacology, Razi Drug Research Center, Iran University of Medical Sciences, Tehran, Iran; 3 Department of Biology, Faculty of Sciences, Islamic Azad University of Qom, Qom, Iran; 4 Avay Mahd Cell Iranian Company, Health Technology Development Center of Qom University of Medical Sciences, Qom, Iran

**Keywords:** Seminal infection, Infertility, Semen parameters

## Abstract

**Background and Objectives:**

There are very few analysis tools to examine seminal fluid specimens, so bacterial infections on male infertility has always been the subject of discussion. These infectious processes lead to deterioration of spermatogenesis, impairment of sperm function, and/or obstruction of the seminal tract. In this study, we aimed at determining the role of bacterial infection on semen parameters including motility, count and normal morphology in infertile male patients.

**Materials and Methods:**

In this cohort study, 150 infertile males having abnormal semen parameters (study group) and 150 healthy fertile males (control group) were included. A total of 300 semen samples were collected after 3 to 5 days of sexual abstinence. Volume, pH, concentration, normal morphology, and motility were evaluated. Samples were seeded using a calibrated loop on agar and EMB plates, which were incubated overnight. The microorganisms were identified by Gram staining technique, catalase and coagulase tests.

**Results:**

The prevalence of seminal infection among infertile males in Qom was 21%. Among these infected samples 61.9%, 14.28%, 14.28% and 9.25% were contaminated with *Staphylococcus aureus*, coagulase negative staphylococci, *Streptococcus* and *Escherichia coli*, respectively. All the identified bacteria except *Streptococcus* caused a significant decrease in sperm concentration. Sperm motility was significantly lower in *E. coli* contaminated samples than in the control group, and the presence of *E. coli* and *S. aureus* led to a decline in normal morphology of the sperms.

**Conclusion:**

Sperm bacterial contamination is quite frequent and could contribute to the deterioration of the sperm quality of infertile males.

## INTRODUCTION

The inability of couples to achieve pregnancy after 12 months of unprotected intercourse, known as infertility, is a worldwide problem, affecting 13% to 15% of all couples (1). Moreover, the worldwide distribution of infertility problems is variable, being higher among underdeveloped than developed countries on account of the availability of related medical services. In more than half of infertile couples, a factor involving the male is responsible for the problem (2). A handful of disorders including varicocele, accessory gland infection, immunological factors, congenital abnormalities, obstructive azoospermia, and endocrine dysfunction are the most common causes of male infertility (3). Infections of the male genitourinary tract account for up to 15% of the cases of male infertility (4). Acute and chronic infections and consequent inflammation of the male reproductive system may compromise sperm cell functions and disrupt spermatogenetic processes (5–7), causing qualitative and quantitative sperm alterations.

Recent studies have shown that the presence of bacteria in semen may compromise sperm quality (7). The bacteria responsible for semen contamination generally originate from the urinary tract of patients, or can be transmitted by a partner via sexual intercourse (8). However, it has been reported that detection of bacteria in semen does not necessarily suggest infection, as bacterial isolates in seminal fluid may also result from sample contamination or colonization of the urethral orifice (9).

Microorganisms can affect the male reproductive function either directly, causing agglutination of motile sperm and thus reducing the ability of acrosome reactions and making alterations in cell morphology, or indirectly through production of reactive oxygen species generated by the inflammatory response to infection (10). The massive infiltration of activated leukocytes into the inflammatory site may be associated with impairment of sperm fertilizing potential due to oxidative, apoptotic, and immune processes (11). Asymptomatic infection caused by *Mycoplasma genitalium* is correlated with male infertility, and antibiotic therapy can improve the semen quality and be used to treat male infertility (12).

The cause–effect relationship among bacterial infections, semen contamination, and male infertility is still being debated. Moreover, the prevalence of bacteria in semen samples of infertile males is similar to that observed in fertile males (13). Thus, the clinical significance of bacteria in semen remains unclear.

This was a case-control study of male patients assessed for couple infertility and fertile individuals. In this study, the effect of infection/colonization of semen on the sperm parameters was investigated. Moreover the prevalence of bacterial infection and its influence on semen parameters including sperm motility, count, and morphology was determined.

## MATERIALS AND METHODS

During 3 months (from June 2016 to September 2016), 300 male individuals, with normal karyotype and without anatomical problems (some of the cases had symptoms of dysuria or painful ejaculation) were referred to Jihad Daneshgahi Infertility Treatment Center (Qom-Iran), a Center that specializes in research and treatment of male infertility and semen analyses, to give a semen analysis. Fertility disorders are the primary reason for patients’ visit to the center. The median duration of infertility among the patient sample (defined as the duration of unprotected intercourse without conception) was 3.75 years (range: 3–7 years). However, we also analyzed the semen samples of males who wished to check their fertility status.

Spermiograms and cultures were performed for each patient to identify bacteria that commonly colonize the male reproductive tract. A control group consisted of semen samples from 100 men with proven fertility who had fathered one or more children during the past 3 years, with normal karyotype and without anatomical problems or infection. Patients and controls provided written informed consent before entering the study. Also, the sample size was selected according to Cochran formula for infertile patients and fertile males as controls.

Semen samples were collected after 3 to 5 days of sexual abstinence. Patients were asked to urinate and wash their hands, penis, and scrotum before ejaculation to avoid possible contamination from urine or external genitalia. Samples were examined after liquefaction for 30 minutes at 37°C at which time volume, pH, concentration, morphology, and motility were assessed according to World Health Organization (WHO) guidelines (14).

Samples were seeded on agar and EMB plates using a calibrated loop, and the plates were incubated overnight at 37°C in normal air with 5% CO_2_. The microorganisms were identified by Gram staining, catalase and coagulase tests.

Data were analyzed by ANOVA and LSD and Fisher’s exact test in SPSS Software Version 11.5. Categorical data were presented as frequency and percentages, while quantitative data were expressed as mean ± standard deviation. The collected data were represented graphically using Microsoft Excel 2010 software. P<0.05 was considered statistically significant. Also, the paint software was used for figure preparation.

## RESULTS

**Fig. 1:**
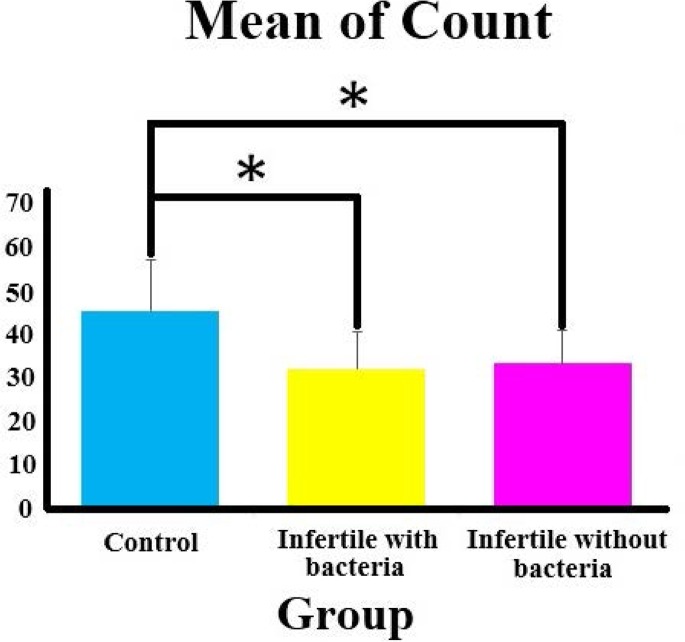
The mean of Count of semen samples in different groups

In each group, semen mean volume, sperm mean count, mean motility, and mean normal morphology of specimen were evaluated according to WHO guidelines (14) and analyzed by light microscopy (Table 1, Fig. 1). Then, 59 infertile males with some symptoms (dysuria or painful ejaculation) were categorized into 2 groups: (1) positive infection (n = 51) and (2) negative infection (n = 8). In the remaining male infertile individuals (n = 241), the symptoms (dysuria or painful ejaculation) were not observed, but presence of bacterial infection was confirmed in 12 cases (Table 2). Out of the 300 patients examined, 63 males (21%) showed the presence of bacterial species in semen samples. Spermioculture in these patients yielded *S. aureus* in 39 samples (61.9%), coagulase negative staphylococci in 9 samples (14.28%), streptococci in 9 samples (14.28%), and *E. coli* in 6 samples (9.52%).

**Table 1:** The mean of evaluated semen parameters in different groups

**Groups**	**Volume (mL)**	**number of sperm/mL×10^6^**	**Motility%**	**normal morphology**
Control	4.08 ± 0.45	45.56 ± 11.65	56.66 ± 8.49	18.46 ± 4.89
Infertile males with bacterial infection	3.14 ± 0.39	32.21 ± 8.41	54.32 ± 4.71	16.25 ± 3.08
Infertile males without bacterial infection	3.74 ± 0.51	33.49 ± 7.45	55.45 ± 6.58	17.01 ± 3.45

**Table 2:** The presence or absence of symptoms in infertile patients with positive and negative bacterial infection

**Groups**	**The presence of symptoms (dysuria or painful ejaculation)**	**The absence of symptoms (dysuria or painful ejaculation)**	**P-value**	**Odds ratio**
Positive infection	51	12		
Negative infection	8	229	0.000	0.008
Total	59	241		

**Fig. 2:**
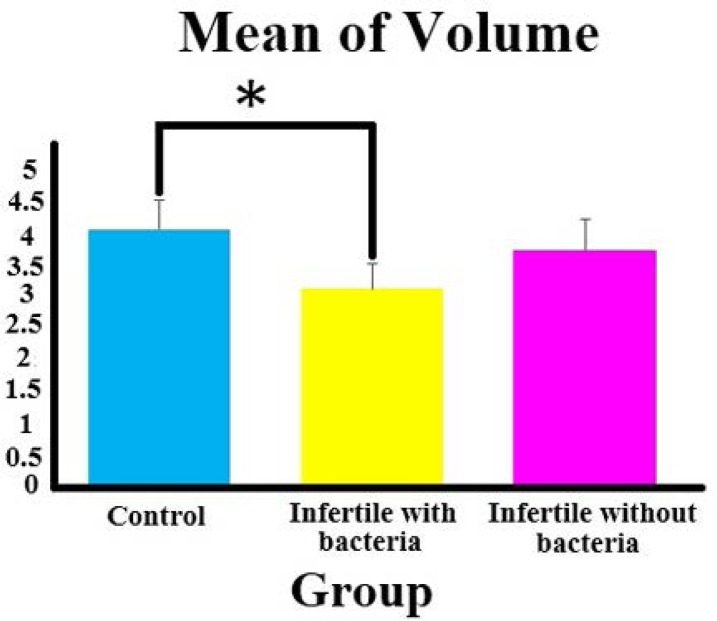
The mean of volume of semen samples in different groups

The mean sperm count in each group was significantly lower than that determined in controls (p>0.05) (Table 3). In the group of infertile males with negative bacterial infection, the sperm volume was also decreased although it was not statistically significant (p>0.05) (Table 3 and Fig. 2).

**Table 3:** Sperm (count, volume, normal morphology) analysis of ejaculates from healthy males and infertile patients with positive and negative bacterial infection. P value ≤ .05 shows a statistically significant difference.

	**Control**				**Infertile without bacteria**				**Infertile with bacteria**			
**Control**	**Count**	**Volume**	**normal morphology**	**Motility**	**Count**	**Volume**	**normal morphology**	**Motility**	**Count**	**Volume**	**normal morphology**	**Motility**
	-	-	-	-	0.456	0.009	0.541	0.418	0.032	0.003	0.621	0.492
Infertile without bacteria	0.456	0.009	0.541	0.418	-	-	-	-	0.046	0.3	0.652	0.568
Infertile with bacteria	0.032	0.003	0.621	0.492	0.046	0.3	0.652	0.568	-	-	-	-

**Fig. 3:**
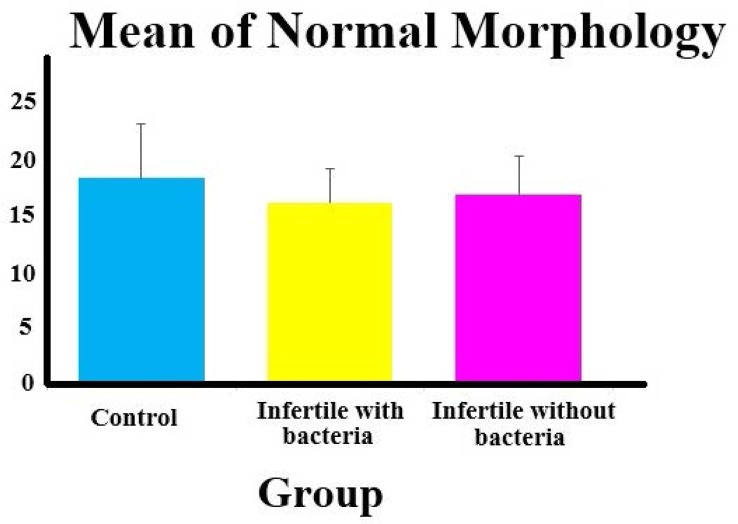
The mean of normal morphology of semen samples in different groups

**Fig. 4:**
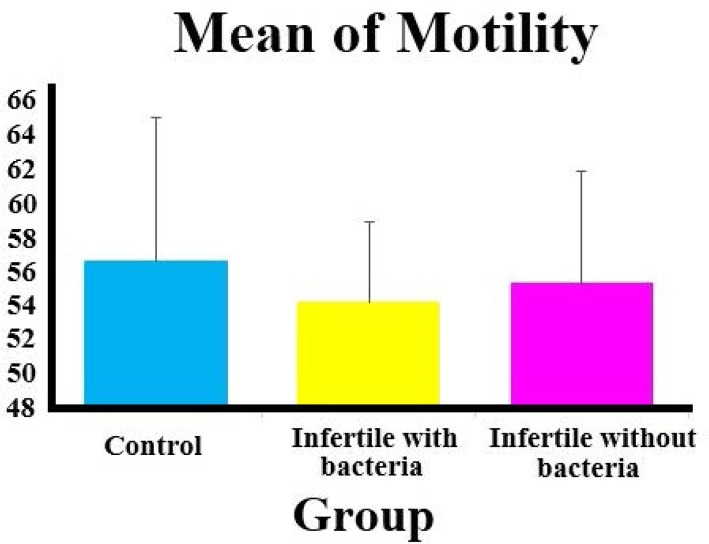
The mean of motility of semen samples in different groups

## DISCUSSION

Semen that passes through the genital tract is routinely contaminated with Gram positive cocci, such as staphylococci and streptococci (15). Infections of *S. aureus* in male reproductive system can lead to the decrease of sperm motility, which may be associated with the *Staphylococcus* complement inhibitor encoded by *scn* gene (16). It is generally accepted that *S. aureus*, which is coagulase positive, is pathogenic and needs special attention, as its persistence can cause damage and loss of germinating host cells. According to Bukharin et al. (2000), opportunistic microorganisms cause classical infections of the urogenital tract and subclinical reproductive tract infections (17).

Many studies have examined the impact of genital tract infections and bacterial semen contamination in male fertility; however, the putative detrimental effect of bacteria on the sperm quality is still controversial (18). Sanocka–Maciejewska et al. (2007) have reported that the bacteria most frequently isolated from the genitourinary tracts of males have no effect on semen quality in normozoospermic males; however, in infertile patients with pathological semen parameters, bacteria usually determined a diminished antioxidant capacity of sperm. These findings were in part been confirmed in the present study, while sperm count and volume were significantly decreased in some bacterial contaminated groups (P<0.05).

The prevalence of bacterial contamination in the infertile population who came to Qom Jihad Daneshgahi Infertility Center was 21%, which is consistent with obtained result reported by Abdel Monem et al. (19). Among the bacteria isolates, the highest count was recorded for staphylococci (61.9%), and this result forcefully correlates with previously published reports that found 68%, 41.5%, 77.7%, and 51.16% of infertile male patients have staphylococcal infections, respectively (19–22). These changes may be due to the difference of study region and distribution of this bacterial strain among infertile couples. Our findings revealed that the simple presence of bacteria might alter sperm quality. The mean sperm count in the samples, positive or negative for bacteria, was significantly lower than that observed in controls.

The negative influence of bacteria on sperm count is well known (7, 23). In our study, count was significantly reduced in male infertile individuals. Our results demonstrated that the presence of bacterial infection can affect the volume and count sperm parameters, and this is in agreement with the report of Abdel Monem et al. (19).

Recent studies have hypothesized that the mechanisms of sperm damage caused by bacteria pass through the expression of the adhesive properties of the flagella and pili to mannose receptors (24). The fact that receptors to mannose have also been demonstrated at the surface of human spermatozoa suggests that flagella and pili could play a considerable causative role in sperm damage (25).

The possible mechanisms of sperm damage, in addition to adhesion by flagella and pili, include the production of toxins and metabolic products originating from bacterial proliferation (26).

In this study, it was observed that bacterial infections can affect some sperm quality parameters, such as volume and count, but the definite effect of the bacterial infections on sperm quality parameters is still controversial.

With the adverse effects that the presence of bacteria in seminal fluid poses on semen parameters, such as count and volume, it seems necessary to evaluate infertile males who want to try an infertility treatment cycle.

In conclusion, treating the bacterial infection of semen is highly important and improvement can be achieved after treatment.
